# Determinants of safe delivery utilization among Indonesian women in eastern part of Indonesia

**DOI:** 10.12688/f1000research.23324.2

**Published:** 2020-09-23

**Authors:** Ferry Efendi, Susy Katikana Sebayang, Erni Astutik, Setho Hadisuyatmana, Eka Mishbahatul Mar'ah Has, Heri Kuswanto

**Affiliations:** 1 Faculty of Nursing, Universitas Airlangga, Surabaya, Indonesia; 2 School of Nursing & Midwifery, La Trobe University, Melbourne, Australia; 3 Department of Biostatistics and Population Studies, Faculty of Public Health, Universitas Airlangga, Banyuwangi Campus, Banyuwangi, Indonesia; 4 Research Group for Health and Wellbeing of Women and Children, Faculty of Public Health, Universitas Airlangga, Banyuwangi, Indonesia; 5 Department of Epidemiology, Faculty of Public Health, Universitas Airlangga, Surabaya, Indonesia; 6 Department of Statistics, Institut Teknologi Sepuluh Nopember (ITS), Surabaya, Indonesia

**Keywords:** facility-based delivery, safe delivery, skilled birth delivery.

## Abstract

**Background: **Improving maternal health and reducing maternal mortality are part of the United Nations global Sustainable Development Goals for 2030. Ensuring every woman’s right to safe delivery is critical for reducing the maternal mortality rate. Our study aimed to identify determinants of safe delivery utilization among women in the eastern Indonesia.

**Methods: **This study was cross-sectional and used a secondary data from the 2017 Indonesian Demographic and Health Survey (IDHS). A total of 2,162 women who had their last child in the five years preceding the survey and lived in the eastern part of Indonesia were selected as the respondents. Chi-squared test and binary logistic regression were used to understand the determinants of safe delivery.

**Results: **Higher child rank and interval ≤2 years (OR: 0.30, 95% CI: 0.19-0.47), unwanted pregnancy at time of becoming pregnant (OR: 1.48, 95% CI: 1.05-2.08), richest wealth quintile (OR: 5.59, 95% CI: 3.37-9.30), more than four antenatal care visits (OR: 3.62, 95% CI: 2.73-4.79), rural residence, good composite labor force participation, and a good attitude towards domestic violence were found to be significantly associated with delivery at health facility. Higher child rank and interval ≤2 years (OR: 0.49, 95% CI: 0.29-0.83), husband/partner having completed secondary or higher education (OR: 2.18, 95% CI: 1.48-3.22), being in the richest wealth quintile, and four other factors were found to be significantly associated with the assistance of skilled birth attendants.

**Conclusions: **This research extends our knowledge on the determinants of safe delivery among women in the eastern part of Indonesia. This study revealed that the economic status of household remains an important issue in improving safe delivery among women in eastern part of Indonesia. An open innovation and partnership process to improve safe delivery program that engages the full range of stakeholders should be developed based on economic situation.

## Introduction

Maternal morbidity and mortality is a global health concern ( World Health Organization, 2017). Every day in 2017, around 830 mothers died due to pregnancy and childbirth ( World Health Organization, 2018). The United Nations Sustainable Development Goals set a target to reduce maternal deaths to 70 per 100,000 live births by 2030 ( United Nations, 2015). In Indonesia, the maternal mortality rate is still high, at 305 per 100,000 live births ( BPS, 2015b). A higher rate was found in the eastern part of Indonesia, namely Nusa Tenggara, Maluku, and Papua Island, than in the other islands ( BPS, 2015a). One of the major causes of maternal mortality is haemorrhage, which is followed by eclampsia ( Tejayanti *et al.,* 2012). Safe delivery as the critical policy of making motherhood safer requires skilled birth attendants and delivery at health facilities across the provinces of Indonesia ( Efendi *et al.,* 2019; Kementerian Kesehatan RI, 2014). As an archipelago country, institutional delivery and skilled assistant delivery are still a challenge because of the geographical situation ( Belton *et al.,* 2014; Ministry of Health [MoH], 2012). To increase safe delivery for Indonesian mothers, the government has set a goal to reach 85% of institutional deliveries in 2019 ( Kementerian Kesehatan RI, 2017). Even though the government has not yet set a goal for skilled attendant delivery specifically in this document, it should be assumed that the government demands the highest standard of health attainment.

**Figure 1.**
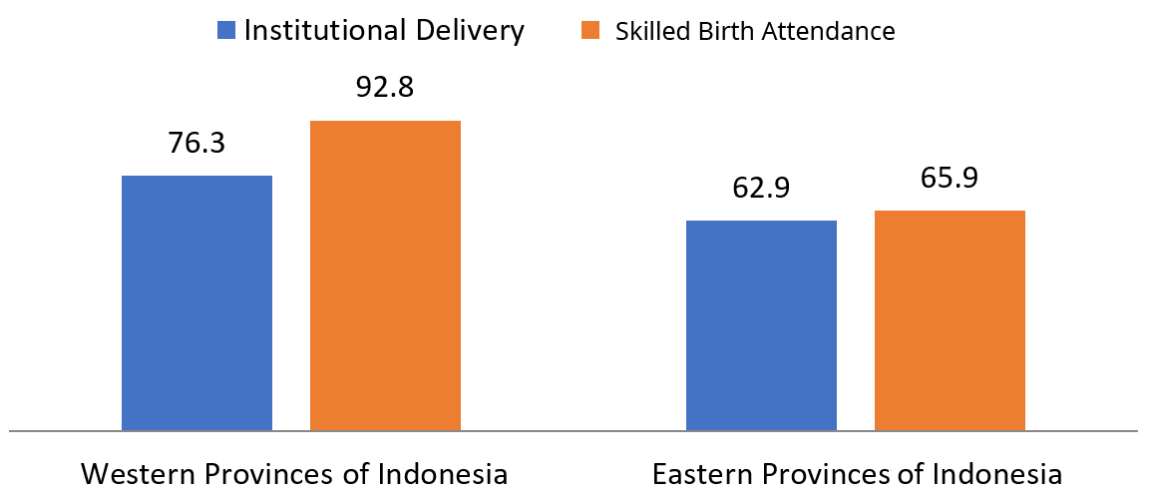
Institutional delivery and skilled birth attendants by western and eastern provinces of Indonesia.

The 2017 IDHS found that there is a gap in coverage of institutional delivery and skilled birth attendants between western provinces and eastern provinces of Indonesia. Eastern provinces of Indonesia, including Bali, Nusa Tenggara Island, Sulawesi Island, Maluku, and Papua Island, have not reached 70% coverage of safe delivery in either institutional delivery or skilled assistant delivery, as depicted in Figure 1 ( BPS *et al.,* 2018).

Studies that examine safe delivery have been conducted in some countries. A study conducted in Ethiopia found that residence, religion, educational level, age at first pregnancy, parity, and antenatal care (ANC) attendance have a significant association with safe delivery service utilization ( Abera *et al.*, 2011). Another study conducted in Tanzania reported that in addition to socio-demographic factors, women’s empowerment status contributed to the decision to give birth with a health professional ( Shimamoto & Gipson, 2015). In a similar vein, studies in 13 sub-Saharan African countries found that living conditions and women’s autonomy are key factors of maternal healthcare utilization ( Iacoella & Tirivayi, 2019). In Indonesia, a study about facility-based childbirth found that educational level, place of residence, working status, involvement in decision-making, economic status, and ANC visits are significantly associated with health facility delivery among women ( Efendi *et al.,* 2019). Furthermore, the gap in age and education between a woman and her husband/partner, women’s self-esteem, age at first marriage, and age at pregnancy were found have a high association with institutional delivery among Indonesian women ( Kurniati *et al.,* 2018).

The gap in coverage of safe delivery, including institutional delivery and skilled birth attendants, in western and eastern provinces of Indonesia should be resolved to attain the Sustainable Development Goals by 2030. Particularly in eastern part of Indonesia, safe delivery, both delivery at health facilitites and delivery assistance by skilled birth attendants remain a critical issue, we hypothesize that determinants associated with safe delivery utilization operate in a fundamentally different way among Indonesian women in this region. We tested this hypothesis using data from the Indonesia Demographic and Health Survey (IDHS) in 2017. Understanding the key factors related to safe delivery is key to prevent maternal morbidity and mortality in Indonesia. Therefore, this study aimed to determine safe delivery utilization among Indonesian women in the eastern part of Indonesia.

## Methods

### Ethical statement

IDHS ethical clearance was obtained from the Inner City Fund (ICF) International. For this study, permission to use the data was obtained from ICF International. This study used existing IDHS data and re-analysis was done under the original consent provided by the participants. Thus, no further consent was obtained from the participants.

### Data source

This was an analytical cross-sectional study that used data from the 2017 IDHS. The 2017 IDHS was conducted in 34 provinces in Indonesia from July to December 2017 by the Central Statistics Agency (BPS), National Population and Family Planning Board (BKKBN), and the Ministry of Health with technical help from ICF. The Individual Recode (IR) dataset was downloaded from www.dhsprogram.com after completing registration.

### Sample size and sampling

This survey covered 1,970 census blocks in urban and rural areas. In the 2017 IDHS, a total of 49,627 women finished the survey from all 34 provinces in Indonesia. DHS sample designs were two-stage probability samples drawn from an existing sample frame. The sampling frame of the 2017 IDHS was the master sample of Census Blocks from the latest population census. The two-stage cluster sampling was used to select the respondents. The first stage was the selection of number of census blocks by systematic sampling proportional to size. In the second stage, 25 ordinary households were taken from the listing. All women aged 15–49 years in the households were eligible for interview. Interviews were performed as privately as possible with a detailed manual as reported by ICF ( ICF Macro, 2020).

The inclusion criteria for this study were women aged 15–49 years who had their last child in the five years preceding the survey and lived in the eastern provinces of Indonesia. For the purpose of analysis, we divided Indonesia into two greater parts, western and eastern, based on the geographical location. The eastern provinces included Bali, West Nusa Tenggara, East Nusa Tenggara, North Sulawesi, Central Sulawesi, South Sulawesi, Southeast Sulawesi, Gorontalo, West Sulawesi, Maluku, North Maluku, West Papua and Papua. In total, survey data from 2,162 women meeting the criteria were accessed for this study’s analysis.

### Variables

The dependent variables in this study were place of delivery and type of assistance at delivery. Place of delivery was divided into two categories: health facility and non-health facility. Health facility delivery or institutional delivery is delivery that is carried out at a heath facility, including public health centers, clinics or maternity homes, and hospitals. The type of assistance at delivery variable was also divided into two categories: skilled birth attendants and unskilled birth attendants. Skilled birth attendants is defined as birth delivered with the assistance of skilled providers such as general practitioners, obstetricians, midwives, and skilled nurses ( Croft *et al.*, 2018).

There were several independent variables in this study. Age difference between man and woman was divided into four categories: woman older than man, 0–4 years younger, 5–7 years younger, and >7 years younger. Birth rank and interval was divided into five categories: second or third child with interval >2 years; first birth, second or third child with interval ≤2 years; fourth or higher child with interval >2 years; and fourth or higher child with interval ≤2 years. Planning status of births, women who had a birth or several births in the five years prior to their interview were asked whether the pregnancy had been wanted at the time it occurred (wanted then) or whether it had been wanted but had occurred sooner than wanted (wanted later), or whether the woman had wanted no further children at the time (unwanted/no more). Husband/partner’s education attainment was divided into three categories: incomplete primary education/none, complete primary or some secondary, and completed secondary or higher. Husband/partner’s occupation was divided into two categories: agricultural and non-agricultural. Wealth quintile was categorized as poorest, poorer, middle, richer, and richest ( Rutstein & Johnson, 2004). Number of household members was divided into two categories: households that have less than four members and households with four or more members. Number of ANC visits was categorized as less than four times and four times or more. Covered by health insurance was divided into two categories: yes and no. Residence was categorized as urban and rural. Women’s empowerment variables, including composite labor force participation, attitude towards domestic violence (wife-beating), decision-making power, and women’s knowledge level, were divided into three categories: poor, moderate, and good. The women’s knowledge level variable was a composite of educational level and access to media. Further details on how these variables were assessed can be found in study as conducted by Sebayang *et al*. (2019).

### Data analysis

The determinants of safe delivery were analyzed using a Chi-square test and binary logistic regression. Both analyses were performed in Stata version 16. The variables were significant at a p-value of 0.05, and the strength of the association was assessed using odds ratio (OR) with a 95% confidence interval (CI).

## Results

Among the women who were included in this study, 71.6% used a health facility and 86.2% were assisted by a skilled birth attendants at their last birth. The majority of the respondents are 0–4 years younger than their husband (41.2%), from the poorest wealth quintile (41.8%), have four or more members in the household (87.6%), are covered by health insurance (64.3%), and live in a rural residence (66.7%). Concerning the husband/partner’s education and occupation, 47.3% have completed secondary or higher education and more than half work in an agricultural occupation (54.1%). For almost half the respondents, their last child was a second or third child with an interval more than two years (44.9%). The majority of respondents had more than four ANC visits (88.4) and their pregnancy was wanted when they became pregnant (82.1%). In terms of women’s empowerment, most respondents have good composite labor force participation (35.7%), a moderate attitude towards domestic violence (34.8%), poor decision-making power (35.3%), and a poor level of knowledge (34.6%). Details about the descriptive characteristics of the respondents are shown in Table 1.

**Table 1.** Characteristics of the respondents regarding determinants of safe delivery utilization among Indonesian women in eastern part of Indonesia (n=2,162).

Variable	n	%
Place of delivery		
Non-health facility	615	28.4
Health facility	1,547	71.6
Type of assistance at delivery		
Unskilled birth attendants	298	13.8
Skilled birth attendants	1,864	86.2
Age difference between man and woman		
Woman older than man	450	20.8
0–4 years	890	41.2
5–7 years	434	20.1
>7 years	388	17.9
Birth rank and interval		
Second or third child, interval >2 years	970	44.9
First birth	603	27.9
Second or third child, interval ≤2 years	131	6.0
Fourth or higher child, interval >2 years	384	17.8
Fourth or higher child, interval ≤2 years	74	3.4
Planning status of births		
Then	1,774	82.1
Later	246	11.4
No more	142	6.5
Husband/partner’s education attainment		
Incomplete primary education/none	299	13.8
Completed primary or some secondary	841	38.9
Completed secondary or higher	1,022	47.3
Husband/partner’s occupation		
Agricultural	1,169	54.1
Non-agricultural	993	45.9
Wealth quintile		
Poorest	903	41.7
Poorer	454	21.0
Middle	315	14.6
Richer	253	11.7
Richest	237	11.0
Number of household members		
<4	268	12.4
≥4	1,894	87.6
Number of antenatal care visits		
<4	251	11.6
≥4	1,911	88.4
Covered by health insurance		
No	772	35.7
Yes	1,390	64.3
Place of residence		
Urban	719	33.3
Rural	1,443	66.7
Labor force participation		
Poor	692	32.0
Moderate	698	32.3
Good	772	35.7
Attitude toward domestic violence		
Poor	674	31.2
Moderate	753	34.8
Good	735	34.0
Decision-making power		
Poor	764	35.3
Moderate	703	32.5
Good	695	32.1
Women’s knowledge level		
Poor	748	34.6
Moderate	714	33.0
Good	700	32.4

In the bivariate analysis, most of the variables showed a significant association with a p-value of 0.05 with both outcomes: place of delivery and type of assistance at delivery. For the place of delivery outcome, three variables have a p-value of more than 0.05 (planning status of births, number of household members, decision-making power), while for the type of assistance at delivery outcome, four variables were not significant (age difference between man and woman, planning status of births, number of household members, decision-making power). Details about the bivariate analysis are shown in Table 2 and Table 3.

**Table 2.** Bivariate analysis of women’s characteristics and place of delivery outcome.

Variable	Non-health facility		Health facility		X ^2^
	N	%	N	%	
Age difference between man and woman					
Woman older than man	117	26.1	333	73.9	17.23 **
0–4 years	262	29.4	628	70.6	
5–7 years	108	24.8	326	75.2	
>7 years	127	32.8	261	67.2	
Birth rank and interval					
Second or third child, interval >2 years	241	24.9	729	75.1	200.57 ***
First birth	124	20.6	479	79.4	
Second or third child, interval ≤2 years	48	36.8	83	63.2	
Fourth or higher child, interval >2 years	156	40.6	228	59.4	
Fourth or higher child, interval ≤2 years	45	60.2	29	39.8	
Planning status of births					
Then	513	28.9	1261	71.1	3.51
Later	68	27.8	178	72.2	
No more	34	23.9	108	76.1	
Husband/partner’s education attainment					
Incomplete primary education/none	143	47.9	156	52.1	233.59 ***
Completed primary or some secondary	277	32.9	564	67.1	
Completed secondary or higher	194	19.0	828	81.0	
Husband/partner’s occupation					
Agricultural	423	36.2	746	63.8	162.94 ***
Non-agricultural	191	19.2	802	80.8	
Wealth quintile					
Poorest	423	46.8	480	53.2	607.20 ***
Poorer	105	23.2	349	76.8	
Middle	45	14.3	270	85.7	
Richer	29	11.3	224	88.7	
Richest	13	5.6	224	94.4	
Number of household members					
<4	67	25.2	201	74.8	3.40
≥4	547	28.9	1,347	71.1	
Number of antenatal care visits					
<4	153	61.6	98	38.4	328.54 ***
≥4	461	24.1	1,450	75.9	
Covered by health insurance					
No	242	31.3	530	68.7	10.16 *
Yes	373	26.8	1,017	73.2	
Residence					
Urban	88	12.2	631	87.8	296.56 ***
Rural	527	36.5	916	63.5	
Labor force participation					
Poor	248	35.8	444	64.2	123.78 ***
Moderate	221	31.7	477	68.3	
Good	145	18.8	627	81.2	
Attitude toward domestic violence					
Poor	237	35.1	437	64.9	46.49 ***
Moderate	194	25.8	559	74.2	
Good	184	25.0	551	75.0	
Decision making power					
Poor	218	28.5	546	71.5	0.64
Moderate	195	27.7	508	72.3	
Good	202	29.0	493	71.0	
Women’s knowledge level					
Poor	290	38.8	458	61.2	145.48 ***
Moderate	187	26.2	527	73.8	
Good	137	19.6	563	80.4	

*p<0.05; **p<0.01; ***p<0.001

**Table 3.** Bivariate analysis of women’s characteristics and type of assistance at delivery.

Variable	Unskilled birth attendants		Skilled birth attendants		X ^2^
	N	%	N	%	
Age difference between man and woman					
Woman older than man	56	12.4	394	87.6	
0–4 years	125	14.0	765	86.0	7.55
5–7 years	54	12.5	380	87.5	
>7 years	63	16.3	325	83.7	
Birth rank and interval					
Second or third child, interval >2 years	113	11.6	857	88.4	
First birth	53	8.8	550	91.2	136.16 ***
Second or third child, interval ≤2 years	22	16.6	109	83.4	
Fourth or higher child, interval >2 years	87	22.7	297	77.3	
Fourth or higher child, interval ≤2 years	24	31.9	50	68.1	
Planning status of births					
Then	254	14.3	1,520	85.7	5.43
Later	29	11.8	217	88.2	
No more	15	10.5	127	89.5	
Husband/partner’s education attainment					
Incomplete primary education/none	106	35.4	193	64.6	367.17 ***
Completed primary or some secondary	130	15.5	711	84.5	
Completed secondary or higher	61	6.0	961	94.0	
Husband/partner’s occupation					
Agricultural	229	19.6	940	80.4	155.85 ***
Non-agricultural	69	6.9	924	93.1	
Wealth quintile					
Poorest	243	26.9	660	73.1	
Poorer	39	8.6	415	91.4	509.25 ***
Middle	11	3.4	304	96.6	
Richer	3	1.3	250	98.7	
Richest	2	0.7	235	99.3	
Number of household members					
<4	30	11.3	238	88.7	3.24
≥4	267	14.1	1,627	85.9	
Number of antenatal care visits					
<4	102	40.5	149	59.5	364.74 ***
≥4	197	10.3	1,714	89.7	
Covered by health insurance					
No	121	15.7	651	84.3	8.03 *
Yes	177	12.7	1,213	87.3	
Residence					
Urban	38	5.3	681	94.7	139.61 ***
Rural	260	18.0	1,183	82.0	
Labor force participation					
Poor	125	18.0	567	82.0	
Moderate	119	17.0	579	83.0	95.35 ***
Good	56	7.2	716	92.8	
Attitude toward domestic violence					
Poor	111	16.5	563	83.5	20.25 **
Moderate	106	14.1	647	85.9	
Good	80	10.9	655	89.1	
Decision-making power					
Poor	119	15.6	645	84.4	7.34
Moderate	92	13.1	611	86.9	
Good	86	12.4	609	87.6	
Women’s knowledge level					
Poor	180	24.1	568	75.9	231.52 ***
Moderate	74	10.4	640	89.6	
Good	43	6.1	657	93.9	

*p<0.05; **p<0.01; ***p<0.001

In the binary logistic regression analysis, delivery at a health facility was associated with several variables. Women who lived in a rural residence [AOR=0.49; 95% CI=0.36-0.66] were less likely to deliver in a health facility compared to women who lived in urban residence. A similar result was found for women whose last child was a fourth or higher child with an interval of two years or under [AOR=0.30; 95% CI=0.19-0.47]. Women from the richest wealth quintile family and those who had four or more ANC visits were five [AOR=5.59; 95% CI=3.37-9.30] and three times more likely to deliver in a health facility [AOR=3.62; 95% CI=2.73-4.79], respectively, compared to their reference.

Women who have good composite labor force participation [AOR=1.47; 95% CI=1.15-1.89] and a moderate attitude towards domestic violence [AOR=1.38; 95% CI=1.10-1.73] were more likely to deliver in a health facility. Women whose pregnancy was unwanted when they became pregnant [AOR=1.48 95% CI=1.05-2.08] were also more likely to deliver in a health facility. Details about the binary logistic regression analysis with a place of delivery outcome are shown in Table 4.

**Table 4.** Binary logistic regression analysis with a place of delivery outcome.

Variable	AOR	CI	
		Lower	Upper
Age difference between man and woman			
Woman older than man	Ref		
0–4 years	0.88	0.69	1.11
5–7 years	1.14	0.88	1.46
>7 years	0.96	0.73	1.26
Birth rank and interval			
Second or third child, interval >2 years	Ref		
First birth	1.37 **	1.09	1.71
Second or third child, interval ≤2 years	0.60 **	0.43	0.84
Fourth or higher child, interval >2 years	0.66 ***	0.52	0.82
Fourth or higher child, interval ≤2 years	0.30 ***	0.19	0.47
Planning status of births			
Then	Ref		
Later	1.01	0.77	1.32
Unwanted	1.48 *	1.05	2.08
Husband/partner’s education attainment			
Incomplete primary education/ none	Ref		
Completed primary or some secondary	1.23	0.94	1.60
Completed secondary or higher	1.26	0.94	1.69
Husband/partner’s occupation			
Agricultural	Ref		
Non-agricultural	1.17	0.93	1.47
Wealth quintile			
Poorest	Ref		
Poorer	1.94 ***	1.53	2.47
Middle	2.85 ***	2.06	3.94
Richer	3.23 ***	2.25	4.64
Richest	5.59 ***	3.37	9.30
Number of household members			
<4	Ref		
≥4	1.02	0.75	1.39
Number of antenatal care visits			
<4	Ref		
≥4	3.62 ***	2.73	4.79
Covered by health insurance			
No	Ref		
Yes	1.15	0.95	1.38
Place of residence			
Urban	Ref		
Rural	0.49 ***	0.36	0.66
Labor force participation			
Poor	Ref		
Moderate	1.14	0.93	1.40
Good	1.47 **	1.15	1.89
Attitude toward domestic violence			
Poor	Ref		
Moderate	1.38 **	1.10	1.73
Good	1.33 *	1.04	1.69
Decision-making power			
Poor	Ref		
Moderate	0.85	0.69	1.06
Good	0.84	0.66	1.06
Women’s knowledge level			
Poor	Ref		
Moderate	1.13	0.92	1.40
Good	1.17	0.93	1.47

*p<0.05; **p<0.01; ***p<0.001

AOR, adjusted odds ratio; CI, confidence interval.

According to the type of assistance at delivery outcome, women whose last child was a fourth or higher child with an interval of two years or under [AOR=0.49; 95% CI=0.29-0.83] were less likely to deliver with an assistance of skilled birth attendants. Women whose husband completed secondary or higher education were two times [AOR=2.18; 95% CI=1.48-3.22] more likely to deliver with an assistance of skilled birth attendants. Likewise, women who had four or more ANC visits and were from the richest wealth quintile were three [AOR=3.83; 95% CI=2.77-5.30] and 15 times [AOR=15.69; 95% CI= 5.53-44.50] more likely to be helped by a skilled birth attendants, respectively.

Women whose husband worked in a non-agricultural occupation [AOR=1.35; 95% CI=1.00-1.81] were more likely to deliver with a skilled birth attendants. A similar result was found for women with good composite labor force participation [AOR=1.58; 95% CI=1.11-2.26] and a good level of knowledge [AOR=1.76; 95% CI=1.25-2.46] ( Table 5).

**Table 5.** Binary logistic regression analysis with type of assistance at delivery.

Variable	AOR	CI	
		Lower	Upper
Age difference between man and woman			
Woman older than man	Ref		
0–4 years	0.89	0.65	1.22
5–7 years	0.99	0.71	1.36
>7 years	1.01	0.72	1.42
Birth rank and interval			
Second or third child, interval >2 years	Ref		
First birth	1.28	0.93	1.76
Second or third child, interval ≤2 years	0.80	0.48	1.33
Fourth or higher child, interval >2 years	0.73	0.53	1.00
Fourth or higher child, interval ≤2 years	0.49 **	0.29	0.83
Planning status of births			
Then	Ref		
Later	1.12	0.74	1.68
No more	1.58	0.96	2.59
Husband/partner’s education attainment			
Incomplete primary education/ none	Ref		
Completed primary or some secondary	1.90 ***	1.42	2.55
Completed secondary or higher	2.18 ***	1.48	3.22
Husband/partner’s occupation			
Agricultural	Ref		
Non-agricultural	1.35 *	1.00	1.81
Wealth quintile			
Poorest	Ref		
Poorer	2.33 ***	1.70	3.18
Middle	5.14 ***	3.19	8.28
Richer	10.84 ***	5.34	22.01
Richest	15.69 ***	5.53	44.50
Number of household members			
<4	Ref		
≥4	0.87	0.56	1.35
Number of antenatal care visits			
<4	Ref		
≥4	3.83 ***	2.77	5.30
Covered by health insurance			
No	Ref		
Yes	1.19	0.94	1.51
Residence			
Urban	Ref		
Rural	0.84	0.54	1.30
Labor force participation			
Poor	Ref		
Moderate	0.99	0.76	1.29
Good	1.58 *	1.11	2.26
Attitude toward domestic violence			
Poor	Ref		
Moderate	1.24	0.94	1.63
Good	1.30	0.96	1.77
Decision-making power			
Poor	Ref		
Moderate	0.89	0.69	1.14
Good	0.99	0.73	1.34
Women’s knowledge level			
Poor	Ref		
Moderate	1.54 **	1.13	2.11
Good	1.76 **	1.25	2.46

*p<0.05; **p<0.01; ***p<0.001

AOR, adjusted odds ratio; CI, confidence interval.

## Discussion

Delivery was regarded as safe when it was attended by a skilled birth attendant and took place in a health facility. This study found that several variables have a significant association with place of delivery and type of assistance at delivery. Women from the richest wealth quintile were more likely to have a delivery in a health facility than those from the poorest wealth quintile. This finding is consistent with that of a previous study in Indonesia. The wealth index of the household would contribute to the access to health care services, including institutional delivery ( Caulfield *et al.,* 2016; Do *et al.,* 2015; Efendi *et al.,* 2019; Roro *et al.,* 2014). Women from the richest wealth quintile were more likely to have a delivery with a skilled birth attendants than those from the poorest wealth quintile. This finding is consistent with that of a previous study conducted in Bangladesh ( Muhammed *et al.,* 2017). Women from low-income families may find it difficult to pay for a skilled birth attendants, so they prefer to give birth without professional assistance ( Muhammed *et al.,* 2017). Therefore, the coverage of health insurance must be enhanced so that women in all the wealth quintiles can have equal access to health care services.

A higher child rank and interval of ≤2 years was associated with a lower chance of women having a delivery in a health facility and being assisted by a health professional. This result is similar to those of studies conducted in Ethiopia and Nigeria ( Abera *et al.,* 2011; Ononokpono & Odimegwu, 2014). Women with a higher child rank will have more experience with pregnancy and delivery, so they feel that they have the confidence to have a delivery outside a health facility ( Abera *et al.,* 2011). Another argument is that women have limited access to health services due to the burden of their economic situation ( Ononokpono & Odimegwu, 2014). The results for delivery with a skilled birth attendant are similar to those of studies conducted in Sudan and Ethiopia ( Mustafa & Mukhtar, 2015; Wilunda *et al.,* 2015). Women with a higher birth rank tend to rely on their experience from previous pregnancies, believing they already know about childbirth. Consequently, they choose to give birth without professional assistance ( Mustafa & Mukhtar, 2015). Review studies conducted in African countries also highlighted the link between higher parity and lower likelihood of delivery at health facility ( Moyer & Mustafa, 2013). Therefore, health education about safe delivery should prioritize mothers with a high child rank by giving them greater access to free health care services.

Women who had more than four ANC visits during their pregnancy were found to be three times more likely to have a safe delivery. This is consistent with the results of studies conducted in Uganda and Ethiopia ( Abera *et al.,* 2011; Atusiimire *et al.,* 2019). Furthermore, a population-based study conducted in Bangladesh had a similar result, which emphasized the positive effect of the ANC on utilization of delivery at health facility ( Pervin *et al.,* 2012). The ANC can prevent unsafe delivery because it will provide health education for the mother, giving information and recommending the place of delivery according to the mother’s and fetus’s condition ( Atusiimire *et al.,* 2019). Women who had more than four ANC visits during their pregnancy were found to be more likely to have a skilled birth attendant. This is consistent with the result of a study that was conducted in Kenya ( Gitimu *et al.,* 2015). ANC attendance will influence the decision of the mother to have an assisted delivery because the ANC emphasizes the importance of safe delivery ( Gitimu *et al.,* 2015). ANC visits must be optimized for pregnant women so that mothers are more exposed to information about safe delivery. The information that the mother receives will influence the decision on where to deliver the baby. Therefore, a minimum number of ANC visits should be given to all pregnant women so they can monitor the condition of the baby and have more knowledge about safe pregnancy and delivery.

Another finding was that women who wanted no more pregnancies when they became pregnant were more likely to give birth in a health facility. This finding is consistent with that of a study conducted in Egypt ( Marston & Cleland, 2003). However, it is inconsistent with the results of a study conducted in Bangladesh, which showed that women who have an unintended pregnancy were less likely to visit an ANC service and more likely to have a home delivery ( Kamal, 2013). There was no study that explained this issue, as it may be related to the social norms and health system of the country itself. We assume that the desire to limit chidbearing may give sufficient time for women to plan ahead some alternatives including the place of delivery. Therefore, this topic should be analyzed further by considering other variables.

Women from a rural residence were found to be less likely to have a delivery in a health facility. This finding is similar to those of previous studies conducted in Bangladesh and Indonesia ( Efendi *et al.,* 2019; Kamal, 2013; Kenea & Jisha, 2017). Living in an urban residence allows easier access to health facilities than living in rural areas. In addition, access to information is easier in urban areas so information about safe delivery can be spread more easily ( Kamal, 2013). Therefore, the gap between rural and urban areas should be taken into consideration by the government regarding the issue of maternal and child health.

Women who had good composite labor force participation and a good attitude towards domestic violence were more likely to have a delivery in a health facility and be assisted by a health professional. This is consistent with the result of studies conducted in Ethiopia and Bangladesh ( Kamal, 2013; Tiruneh *et al.,* 2017). In Bangladesh, women who were against domestic violence and more independent economically were more likely to have four or more ANC service visits, which may lead the women to have a delivery in a health facility ( Kamal, 2013). Women who had good composite labor force participation and a good knowledge level were more likely to have a delivery with an assistance of skilled birth attendants. This is consistent with the result of studies conducted in Senegal and Tanzania ( Shimamoto & Gipson, 2015). If women have greater empowerment, in terms of knowledge and economic power, this will lead to improvement in their health. They can choose the best for their health, including choosing to have a safe delivery ( Prata *et al.,* 2017). Therefore, gender equality needs to be improved so women can make decisions about their health.

Husband/partner’s education attainment was found to be significantly associated with skilled birth attendants delivery. Women whose husband/partner had completed secondary or higher education were more likely to have a skilled birth attendant. This is consistent with the results of studies conducted in Kenya and Somalia ( Gitimu *et al.,* 2015; Yusuf *et al.,* 2017). Husbands with a higher level of education will have more knowledge about health, including safe delivery ( Kifle *et al.,* 2018). As the head of the household, the husband’s knowledge will affect the reproductive health decisions ( Yusuf *et al.,* 2017). Engagement of the husband in the issue of maternal health should be expanded in all levels of the community.

Women whose husband/partner’s occupation was non-agricultural were found to be significantly more likely to have a delivery with professional assistance. This finding is similar to those of studies conducted in Nigeria and Ethiopia ( Adewemimo *et al.,* 2014; Fekadu & Regassa, 2014). The husband’s occupation will affect the family’s income. If the family income increases, the decision to have a skilled birth attendant will also be affected. In addition, another study conducted in Ethiopia found that women whose husbands work in a non-agricultural occupation tend to use an ANC service, which encourages the decision to give birth with professional assistance ( Tsegay *et al.,* 2013). Therefore, health promotion about safe delivery is important for the husband/partner, especially for husbands/partners whose occupation is agricultural.

## Limitations and strengths

This study used secondary data from the 2017 IDHS, so the selection of the variables was determined by the availability of the data. Another limitation is that some questions in the survey needed respondents to recall what happened five years preceding the survey, so the information may not be precisely stated. In addition to the limitations, however, this study has strengths. The sample of this study was selected using two-stage cluster sampling, so the data were nationally representative. Therefore, the results can provide recommendations for policymakers to develop effective regulation so the coverage gap of safe delivery between western and eastern provinces in Indonesia can be reduced. The practical benefits of this study is to facilitate use of these data for planning, policy-making and program management in the area of maternal health especially the safe delivery.

## Conclusions

Safe delivery was found to be determined by several factors, which reflected the need for multi-stakeholder intervention in increasing the practice of safe delivery across the country. Programmatic and structured policies that target poor women and those with a low education level and encourage husbands/partners’ participation in this issue may help increase the prevalence of safe delivery in the eastern part of Indonesia. This study gives some recommendations to the policymakers, such as health promotion about safe delivery should be prioritized for women who have a high birth rank. Moreover, not only the women, but also their husband/partner should be involved in health education. The coverage of health insurance and health facilities should be enhanced so that everyone can have equal access to health services. Furthermore, the women’s empowerment program should be maximized so that all the women can choose the best for their health.

## Data availability

Data used in this study is available online from the Indonesian 2017 Demographic and Health Survey (DHS) website under the ‘Individual Recode’ section. Access to the dataset requires registration and is granted only for legitimate research purposes. A guide for how to apply for dataset access is available at: https://dhsprogram.com/data/Access-Instructions.cfm.
