# Supplementary material for: Determinants of safe delivery utilization among Indonesian women in eastern part of Indonesia
Source: F1000Res. 2020 Sep 23;9:332. Originally published 2020 May 5. [Version 2] doi: 10.12688/f1000research.23324.2 (PMC7444318; doi:10.12688/f1000research.23324.2)
Supplement: Peer review history

# Peer review: Reviewer response for version 2

**DOI:** 10.5256/f1000research.29415.r71837

**Published:** 2020-09-24

**Authors:** Ryan Michael Flores Oducado

**Affiliations:** 1 College of Nursing, West Visayas State University, Iloilo City, Philippines

I have no further comments to make. The researchers did a good job in revising the article.

Is the work clearly and accurately presented and does it cite the current literature?

Yes

If applicable, is the statistical analysis and its interpretation appropriate?

Yes

Are all the source data underlying the results available to ensure full reproducibility?

Yes

Is the study design appropriate and is the work technically sound?

Yes

Are the conclusions drawn adequately supported by the results?

Yes

Are sufficient details of methods and analysis provided to allow replication by others?

Yes

Reviewer Expertise:

Public Health and Community Health Nursing

I confirm that I have read this submission and believe that I have an appropriate level of expertise to confirm that it is of an acceptable scientific standard.

# Peer review: Reviewer response for version 1

**DOI:** 10.5256/f1000research.25747.r66055

**Published:** 2020-08-21

**Authors:** Asmaa Salah Eldin Mohamed Saleh

**Affiliations:** 1 Community Health Nursing, Faculty of Nursing, Beni Suef University, Beni Suef, Egypt

This is a good written article, but there are little things I think to make it perfect if the author follows it.

 First: in Introduction: the author does not mention study hypotheses. Explain why the current research is important.

 What value does the paper add?

 What practical benefits will it provide?

 Second: Sample and sampling, it is important to add and mention the procedure of taking sampling for this huge sample size.

Is the work clearly and accurately presented and does it cite the current literature?

Yes

If applicable, is the statistical analysis and its interpretation appropriate?

I cannot comment. A qualified statistician is required.

Are all the source data underlying the results available to ensure full reproducibility?

Yes

Is the study design appropriate and is the work technically sound?

Yes

Are the conclusions drawn adequately supported by the results?

Yes

Are sufficient details of methods and analysis provided to allow replication by others?

Yes

Reviewer Expertise:

community health nursing, geriatric health, MCH, health promotion, school health, pediatrics, maternity

I confirm that I have read this submission and believe that I have an appropriate level of expertise to confirm that it is of an acceptable scientific standard.

# Peer review: Reviewer response for version 1

**DOI:** 10.5256/f1000research.25747.r63312

**Published:** 2020-05-26

**Authors:** Ryan Michael Flores Oducado

**Affiliations:** 1 College of Nursing, West Visayas State University, Iloilo City, Philippines

This article looks into the determinants of safe delivery in the eastern part of Indonesia. The report addresses an important topic and contributes to the advancement of knowledge on maternal health and in reducing maternal mortality.

 I only have a few comments regarding the research article:

1. The authors mentioned that the dependent variables were facility-based delivery and assisted delivery. Will it be more appropriate to consider a) place of delivery and b) proportion of women assisted by skilled birth attendants as dependent variables?
2. It was reported in the article that 71.6% used a health facility and 86.2% were assisted by a health professional at their last birth. I wonder, isn’t that health professionals typically assist health facility delivery? What is the reason for the discrepancy?
3. Please clarify women’s knowledge level. This variable pertains to women’s level of knowledge about what?
4. An interesting finding in this study was that women who wanted no more pregnancies when they became pregnant were more likely to give birth in a health facility. The authors mentioned that it might be related to the social norms and health system of the country itself. Can the authors expound on this? Can the researchers offer their plausible explanation of this result?
5. Can the authors restate the conclusions in the abstract? Can household factors be changed to a more encompassing term reflective of the result? Can the facility-based delivery term be deleted and focus on safe delivery as directed in the title of the article?
6. Can the use of secondary data be mentioned in the abstract?

Thank you very much for the opportunity to review this article.

Is the work clearly and accurately presented and does it cite the current literature?

Yes

If applicable, is the statistical analysis and its interpretation appropriate?

Yes

Are all the source data underlying the results available to ensure full reproducibility?

Yes

Is the study design appropriate and is the work technically sound?

Yes

Are the conclusions drawn adequately supported by the results?

Yes

Are sufficient details of methods and analysis provided to allow replication by others?

Yes

Reviewer Expertise:

Public Health and Community Health Nursing

I confirm that I have read this submission and believe that I have an appropriate level of expertise to confirm that it is of an acceptable scientific standard.

# Response

**Published:** 2020-09-04

**Authors:** Ferry Efendi

**Affiliations:** Universitas Airlangga, Indonesia

We thank all reviewers for their generous and positive comments on our manuscript. With regard to their reviews, we have edited the manuscript to respond to their comments. In particular, we have made updates in track changes to indicate changes as suggested. We thank you for the opportunity to submit our revised manuscript.

 Reviewer 1

 This article looks into the determinants of safe delivery in the eastern part of Indonesia. The report addresses an important topic and contributes to the advancement of knowledge on maternal health and in reducing maternal mortality.

 I only have a few comments regarding the research article:

 1. The authors mentioned that the dependent variables were facility-based delivery and assisted delivery. Will it be more appropriate to consider a) place of delivery and b) proportion of women assisted by skilled birth attendants as dependent variables?

 Thank you for these constructive comments, we have changed the terminology into place of delivery (health facility Vs non-health facility) and type of assistance at delivery (skilled birth attendants Vs unskilled birth attendants) as guided by guide to DHS statistics  DHS-7 and your comments.

 2. It was reported in the article that 71.6% used a health facility and 86.2% were assisted by a health professional at their last birth. I wonder, isn’t that health professionals typically assist health facility delivery? What is the reason for the discrepancy?

 Thank you for raising this issue, all delivery in health facility must be assisted by health professionals. However, not all delivery assisted by health professionals occurred in health facility.

 3. Please clarify women’s knowledge level. This variable pertains to women’s level of knowledge about what?

 Thank you very much, we have added the explanation in variables section.  

  4. An interesting finding in this study was that women who wanted no more pregnancies when they became pregnant were more likely to give birth in a health facility. The authors mentioned that it might be related to the social norms and health system of the country itself. Can the authors expound on this? Can the researchers offer their plausible explanation of this result?

 Thank you very much, we have added the explanation in discussion section.

 5. Can the authors restate the conclusions in the abstract? Can household factors be changed to a more encompassing term reflective of the result? Can the facility-based delivery term be deleted and focus on safe delivery as directed in the title of the article?

 Thank you very much, we have revised the abstract.

 6. Can the use of secondary data be mentioned in the abstract?

 Thank you very much, we have added in the abstract section.

# Response

**Published:** 2020-09-04

**Authors:** Ferry Efendi

**Affiliations:** Universitas Airlangga, Indonesia

We thank all reviewers for their generous and positive comments on our manuscript. With regard to their reviews, we have edited the manuscript to respond to their comments. In particular, we have made updates in track changes to indicate changes as suggested. We thank you for the opportunity to submit our revised manuscript.

 Reviewer 2

 1. This is a good written article, but there are little things I think to make it perfect if the author follows it.

 First: in Introduction: the author does not mention study hypotheses. Explain why the current research is important. What value does the paper add? What practical benefits will it provide?

  Thank you very much, we have added on introduction section for the hypothesis and the value. While, we have added the practical benefits on the limitation and strength section.   

 2. Second: Sample and sampling, it is important to add and mention the procedure of taking sampling for this huge sample size.

 Thank you very much, we have added on sample size and sampling section.
